# Evaluation of Health-Related Quality of Life according to Carbohydrate Metabolism Status: A Spanish Population-Based Study (Di@bet.es Study)

**DOI:** 10.1155/2012/872305

**Published:** 2012-07-17

**Authors:** C. Marcuello, A. L. Calle-Pascual, M. Fuentes, I. Runkle, F. Soriguer, A. Goday, A. Bosch-Comas, E. Bordiú, R. Carmena, R. Casamitjana, L. Castaño, C. Castell, M. Catalá, E. Delgado, J. Franch, S. Gaztambide, J. Girbés, R. Gomis, G. Gutiérrez, A. López-Alba, M. T. Martínez-Larrad, E. Menéndez, I. Mora-Peces, E. Ortega, G. Pascual-Manich, G. Rojo-Martínez, M. Serrano-Rios, S. Valdés, J. A. Vázquez, J. Vendrell

**Affiliations:** ^1^Department of Endocrinology and Nutrition, Hospital Clínico San Carlos de Madrid, Profesor Martín Lagos s/n, 28040 Madrid, Spain; ^2^Preventive Medicine Service, Hospital Clínico San Carlos de Madrid, Profesor Martín Lagos s/n, 28040 Madrid, Spain; ^3^Centro de Investigación Biomédica en Red de Diabetes y Enfermedades Metabólicas Asociadas (CIBERDEM), Spain; ^4^Department of Endocrinology and Nutrition, Hospital Regional Universitario Carlos Haya Málaga, Málaga, Spain; ^5^Department of Endocrinology and Nutrition, Hospital del Mar, 08003 Barcelona, Spain; ^6^Institut d'Investigacions Biomèdiques August Pi i Sunyer (IDIBAPS), 08036 Barcelona, Spain; ^7^Laboratorio de Endocrinología, Hospital Clínico San Carlos de Madrid, 28040 Madrid, Spain; ^8^Department of Medicine and Endocrinology, Hospital Clínico Universitario de Valencia, 40010 Valencia, Spain; ^9^Biomedic Diagnostic Centre, Hospital Clínic de Barcelona, 08007 Barcelona, Spain; ^10^Diabetes Research Group, Hospital Universitario de Cruces, UPV-EHU, Barakaldo, Spain; ^11^Public Health Division, Department of Health, Autonomous Government of Catalonia, Barcelona, Spain; ^12^Department of Endocrinology and Nutrition, Hospital Central de Asturias, 33006 Oviedo, Spain; ^13^EAP Raval Sud, Institut Català de la Salut, Red GEDAPS, Primary Care, Unitat de Suport a la Recerca (IDIAP—Fundació Jordi Gol), Barcelona, Spain; ^14^Diabetes Unit, Hospital Arnau de Vilanova, 46015 Valencia, Spain; ^15^Endocrinology and Diabetes Unit, Hospital Clínic de Barcelona, Universitat de Barcelona (UB), 08007 Barcelona, Spain; ^16^Spanish Diabetes Society, Madrid, Spain; ^17^Lipids and Diabetes Laboratory, Hospital Clínico San Carlos de Madrid, Profesor Martín Lagos s/n, 28040 Madrid, Spain; ^18^Canarian Health Service, Tenerife, Spain; ^19^Diabetes National Plan, Ministry of Health, Madrid, Spain; ^20^Department of Endocrinology and Nutrition, Hospital Universitario Joan XXIII, Institut d'Investigacions Sanitaries Pere Virgili, Tarragona, Spain

## Abstract

*Objective*. To evaluate the association between diabetes mellitus and health-related quality of life (HRQOL) controlled for several sociodemographic and anthropometric variables, in a representative sample of the Spanish population. *Methods*. A population-based, cross-sectional, and cluster sampling study, with the entire Spanish population as the target population. Five thousand and forty-seven participants (2162/2885 men/women) answered the HRQOL short form 12-questionnaire (SF-12). The physical (PCS-12) and the mental component summary (MCS-12) scores were assessed. Subjects were divided into four groups according to carbohydrate metabolism status: normal, prediabetes, unknown diabetes (UNKDM), and known diabetes (KDM). Logistic regression analyses were conducted. *Results*. Mean PCS-12/MCS-12 values were 50.9 ± 8.5/ 47.6 ± 10.2, respectively. Men had higher scores than women in both PCS-12 (51.8 ± 7.2 versus 50.3 ± 9.2; *P* < 0.001) and MCS-12 (50.2 ± 8.5 versus 45.5 ± 10.8; *P* < 0.001). Increasing age and obesity were associated with a poorer PCS-12 score. In women lower PCS-12 and MCS-12 scores were associated with a higher level of glucose metabolism abnormality (prediabetes and diabetes), (*P* < 0.0001 for trend), but only the PCS-12 score was associated with altered glucose levels in men (*P* < 0.001 for trend). The Odds Ratio adjusted for age, body mass index (BMI) and educational level, for a PCS-12 score below the median was 1.62 (CI 95%: 1.2–2.19; *P* < 0.002) for men with KDM and 1.75 for women with KDM (CI 95%: 1.26–2.43; *P* < 0.001), respectively. *Conclusion*. Current study indicates that increasing levels of altered carbohydrate metabolism are accompanied by a trend towards decreasing quality of life, mainly in women, in a representative sample of Spanish population.

## 1. Introduction

Diabetes mellitus is a disease that has a serious impact on the public health, owing to its high prevalence and its association with chronic complications [1].

Recently published data in our country indicate an increase in the prevalence of diabetes mellitus, so that the 13.8% of the population over 18  years of age suffers from diabetes, whereas the 30% presents either impaired fasting glucose or impaired glucose tolerance [2]. It induces a considerable economic burden for the national health system and for the society, as well as a detriment on quality of life. Its association to comorbid conditions and complications represents an opportunity to evaluate the influence of a chronic disease on health-related quality of life (HRQOL). Several studies have reported deterioration in the HRQOL of diabetic patients, particularly as far as their physical function and well-being are concerned, but also in terms of mental health [3–7]. Available data in Spain are scarce and they have been obtained in clinic-based studies or in selected populations, and the outcomes have been inconclusive [8–10]. To improve HRQOL of the diabetic patients remains as an important target of the diabetes management. Beyond development of optimal medication, treatment strategies should take HRQOL and psychosocial aspects into account.

In this study, we evaluate the association between the status in carbohydrate metabolism and HRQOL, controlled for socio-demographic and anthropometric variables, such as age, gender, body mass index (BMI), and educational level, in a large representative sample of the Spanish population. Data obtained could be useful to plan patient-based health decisions. The development of appropriate educational interventions could improve patients' ability to control their disease, thus, resulting in an improvement in quality of life. These results should also be used as reference values to compare HRQOL in other chronic diseases and compared across countries.

## 2. Research Design and Methods

### 2.1. Population

The di@betes study is a national, cross-sectional study designed to estimate the prevalence of Diabetes mellitus in Spain. Data collection took place between 2009 and 2010. 

The participants were invited to attend a single examination visit at their health centre. Information was collected using a structured, interviewer-administered questionnaire, followed by a physical examination. Fieldwork was undertaken by seven teams, comprising a nurse and dietician. After the interview, a fasting blood sample was obtained and a standard OGTT was performed. Subjects with baseline capillary blood glucose levels lower than 7.8 mmoL/L and not receiving treatment for diabetes underwent a standard OGTT, obtaining fasting and 2 h venous samples.

Of the eligible adults, 61.7% (9653) were localized, and 55.8% (5728) attended for examination. Of these, 9.9% were excluded by protocol (institutionalized, serious disease, pregnant, or recent delivery), and 5072 completed the study. Response rate of the study was a 55.8%. Differences in age and gender were not found between responders and no responders. A comprehensive description of the methodology has been previously reported [2]. People who did not complete the survey or whose data were incomplete were excluded (*n* = 25). The present study includes 5047 subjects, aged between 18 and 90 years, who responded to the HRQOL questionnaire SF-12 (2162 men and 2885 women, with a mean age of 50.5 ± 17.3 and 50.4 ± 16.8, resp.). 

#### 2.1.1. Assessment of Quality of Life

The SF-12 (version 1), validated for use in the Spanish population, was used [7, 11, 12]. After its application by a trained interviewer, two summary scales are assessed, the PCS-12 and the MCS 12. These scales have been standardized to a mean score of 50 and a standard deviation of 10 in the general population. The MCS and PCS scores range from 0 to 100, with lower scores indicating lower HRQOL. A higher score in the respective summary scales indicates a higher quality of life [12–14]. The questionnaire was administered and supervised by a trained nutritionist (by interview).

Information regarding self-rated health was obtained from the question “Would you say that in general your health is excellent, very good, good, regular or poor?”, which is one of the twelve items included in SF-12.

Carbohydrate status was evaluated by measuring plasma fasting glucose and/or the oral glucose tolerance test, following the 1999 WHO criteria [15]. The population was divided into the following groups: normal carbohydrate metabolism, prediabetes (impaired fasting glucose and/or impaired glucose tolerance), unknown diabetes mellitus (UNKDM), or known diabetes mellitus (KDM) (in the group of KDM the prevalence of type 1 diabetes is expected to account for only 5–10% of the sample, the same percentage described in the literature) [16].

Body mass index (BMI, kg/m^2^) was classified into four groups: normal weight (BMI < 25 kg/m^2^), overweight (BMI between 25 and 29.9 kg/m^2^), obesity (BMI 30–39.9 kg/m^2^), and morbid obesity (BMI ≥ 40 kg/m^2^). Educational level was estimated based on the highest degree completed and divided into four groups: no studies, elementary school, secondary school education, and university degree. The duration of diabetes mellitus was divided into 3 categories: <5 years, between 5 and 15 years, and >15 years.

The presence of chronic complications was defined as self-reported history of cardiac complications (angina pectoris or myocardial infarction), stroke or circulatory disorders of the legs.

### 2.2. Statistical Study

Qualitative variables were summarized by their frequency distribution as well as quantitative variables by their mean and standard deviation (mean ± SDM). To compare the continuous variables PCS 12 and MCS 12 between more than two groups, the one-way analysis of variance (ANOVA) for quantitative normally distributed variables was used. If a significant difference between the groups in ANOVA was obtained, the statistical significance of multiple comparisons was evaluated by using a Bonferroni correction method.

Associations between quality of life (PCS and MCS) and glucose metabolism status were evaluated by fitting logistic regression analysis. PCS and MCS were used as dependent variables, categorized by the sample's median. Three models are reported: the first one shows the crude rates of association, the second one reports measurements adjusted for age, and the third one shows data adjusted for age, BMI, and educational level. The three models are stratified by gender. Moreover, the effect of gender in the association between glucose metabolism and quality of life was evaluated by introducing this term of interaction into the models.

Null hypothesis was rejected by a type I error minor than 0.05 (*α* < 0.05). Process and analysis of the data were performed using the Statistical Package SPSS version 15.0 for Windows (SPSS, Chicago, IL, USA).

## 3. Results

The PCS 12 value was 50.9 ± 8.5 and the MCS 12 was 47.6 ± 10.2 (mean ± SDM). To the question “How would you define your general health?” 2.3% of the population declared having excellent health, 9% very good, 62.5% good, 22.9% regular, and 3.3% poor health.

Men had higher scores than women in both PCS 12 (51.8 ± 7.2 versus 50.3 ± 9.2; *P* < 0.001) and MCS 12 (50.2 ± 8.5 versus 45.5 ± 10.8; *P* < 0.001).

The scores for PCS 12 and MCS 12 according to age, BMI, educational level, and status of carbohydrate metabolism, stratified by gender, are shown in Table 1.

A progressive deterioration of PCS 12 was observed, as major alterations in carbohydrate metabolism appeared but differences in the mental scale were not found in men.

After adjusting for age, BMI, and educational level (using a logistic regression model), this study found that physical health was considerably lower in people with KDM (men and women) when compared to the population with a normal glucose metabolism. The odds ratio for a PCS-12 score below the median was 1.62 (CI 95%: 1.2–2.19; *P* < 0.002) for men with KDM and 1.75 for women with KDM (CI 95%: 1.26–2.43; *P* < 0.001), respectively (Table 2). Worse HRQOL was defined as having a score inferior than median value, taking in mind that no optimal cut point to differentiate between bad and good quality of life has been established for Spanish population. Median values for PCS 12 and MCS 12 were 54.46 and 50.49, respectively. No differences in mental health were found between groups.

There was no statistically significant interaction between gender and glucose metabolism in any of the models.

In the KDM group, we evaluated the influence of the treatment with insulin, duration of diabetes, and the presence of macrovascular complications in quality of life. PCS 12 was lower in insulin-treated patients as compared with untreated diabetic patients (42.7 ± 11.3 versus 48.2 ± 9.7; *P* < 0.001), in the group of patients who referred at least one chronic complication (43.2 ± 11.0 versus 47.7 ± 9.9; *P* < 0.001), and in diabetes of more than 15 years of duration as compared to those with <5 years length (44.7 ± 10.3 versus 48.1 ± 10.2; *P* < 0.04). Differences in mental scales were not found.

## 4. Discussion

This study estimates differences in HRQOL according to status in carbohydrate metabolism, stratified by several demographic factors, for the first time in a large and representative sample of the Spanish population.

Taking into account the influence of anthropometric and demographic factors, gender is one of the most important factors when studying HRQOL. As expected, women referred a poorer HRQOL than men, regardless of age range or BMI status, in both physical and mental scales. This finding is consistent with previous research [7–9, 17].

A remarkable decrease in the physical scales was observed in the older population, which could be explained by the loss of physical abilities associated with ageing. However, in the mental scales a stabilization or slight improvement was found in the elderly. It has been hypothesized that older people may achieve greater emotional control responsiveness to negative emotions. These findings are consistent with data previously reported the in Spanish population [7–9]. Body weight is one of the modifiable risk factors that could affect HRQOL. Ford et al. [18] found that physical function was more strongly affected than mental function in obese patients. Our study showed similar findings in men, although women showed deterioration in both scores, indicating once more that the way in which HRQOL is reported is highly influenced by gender.

This study found a decrease in the physical score associated with more severe alterations in carbohydrate metabolism. This trend is observed in the mental sphere as well, but only in women. Unexpectedly, after adjusting for different confounding factors, no differences were found between the normal group and the UNKDM population. These data could suggest that the diagnosis of diabetes mellitus and the consequent modifications in life style and/or treatment would suppose a negative impact, modifying the perceived HRQOL of patients.

SF 12 scores previously reported in a German cohort of patients with diabetes are lower than those reported in this study [19] (MCS 12 was 45.8 ± 10.3 and PCS 12 was 38.9 ± 9.8). However, in this paper, 36.0% of the participants had one or more missing items in the SF-12 questionnaires, and to deal with the missing data, they applied a modified regression estimation method. Besides, people included in the study were older (aged 50 to 74 years) than those included in our sample. Similarly, another study [17] carried out in German population also found worse physical quality of life in diabetic population, reporting slightly lower PCS 12 values than our study, but higher MCS 12 values. Again, population in this study was older than in our study (58.8 versus 50.4 years old), fact that could explain the discordance. In addition, the questionnaire in our study was applied by a trained interviewer in order to avoid “missing values.”

According to our data, the presence of chronic complications, the treatment with insulin, and a prolonged course of the disease (over 15 years) decrease physical HRQOL in KDM. The presence of complications is probably the most important factor influencing the HRQOL of diabetic patients [20]. Duration of the disease has a variable effect and might not be such an important factor [3]. Regarding the effects of treatment, the results obtained so far are not conclusive [4, 18, 20, 21]. The present study found lower scores in the insulin-treated group. Unfortunately, multivariate analyses were not done because of small sample size.

Several studies that evaluate HRQOL in subjects with diabetes have been recently published in the Spanish population [22, 23]. In the first study [22], the variables that determined a poorer perception of HRQL among diabetic patients were female gender, older age, obesity, lack of physical exercise, coexistence of depression, and cerebrovascular diseases. The other study [23] reported that the variables associated with an increased risk of self-rated fair or poor health in diabetic patients were age 54–64 years or ≥65 years, presence of comorbidity, female sex, lower educational level, obesity, and no physical activity. Current study shows the OR for having a PCS-12 score below the median, adjusted for age, BMI, and educational level. After adjusting for all these factors, the group of KDM, both men and women, has considerably worse physical quality of life.

The present study has some limitations. Unfortunately HbA1c data were not assessed and therefore it is not possible to estimate HRQOL as a function of glycemic control.

Another possible bias is that the institutionalized population was not included in the sample. This group could have a worse health status than the noninstitutionalized one. 

In summary, to our knowledge, this study is the largest report of HRQOL stratified by carbohydrate metabolism status in a representative sample of the Spanish population. It provides a comprehensive insight into objective and subjective factors that may affect HRQOL in patients with diabetes. Data obtained could be helpful in establishing health programs in diabetic populations to evaluate more precisely self-perception of health or creating educational projects to improve psychological well-being. It will also be useful for comparing data obtained from patients with other chronic diseases and across countries.

## Figures and Tables

**Table 1:** PCS 12 and MCS 12 scores stratified by age, BMI, educational level, and glucose metabolism status.

	*N*	PCS 12 score		MCS 12 score	
	M/W	M	W	M	W
Age (years)					
18–30	305/367	54.9 ± 4.7	53.5 ± 6.5	49.5 ± 8.2	46.1 ± 9.9
31–45	597/849	53 ± 6.1	52.9 ± 6.9	49.5 ± 8.7	45.9 ± 10.2
46–60	575/814	51.5 ± 7.3	50.3 ± 9.2	49.7 ± 8.9	44.9 ± 11.4
61–75	493/605	50.6 ± 7.9	47.9 ± 1	51.8 ± 8.4	45.3 ± 11.4
≥76	192/250	47.7 ± 9.1	43.3 ± 11.8	51.9 ± 7.5	46.7 ± 11.2
SS: ANOVA		*P* < 0.0001	*P* < 0.0001	*P* < 0.0001	NS
BMI (kg/m^2^)					
<25	442/1034	52.9 ± 6.9	52.8 ± 7	49.8 ± 8.9	47.2 ± 9.6
25–29	1027/980	52.4 ± 6.6	50.6 ± 9	50.3 ± 8.2	45 ± 11.2
30–39	638/767	50.6 ± 7.8	47.8 ± 10.3	50.5 ± 8.9	44.7 ± 11.4
≥40	46/91	46.1 ± 10.2	42 ± 12	50.4 ± 9.4	40.5 ± 13.5
SS: ANOVA		*P* < 0.0001	*P* < 0.0001	NS	*P* < 0.0001
Educational level					
No studies	254/404	49.8 ± 8.7	45.6 ± 10.9	50.4 ± 8.8	44.1 ± 11.9
Elementary	755/1082	51.2 ± 7.6	49.4 ± 9.8	50.5 ± 8.9	44.8 ± 11.5
Secondary education	815/950	52.4 ± 6.9	52.1 ± 7.6	50 ± 8.7	46.1 ± 10.1
University degree	338/448	53.6 ± 5.3	53.2 ± 6.9	50.3 ± 7.2	47.6 ± 9.4
SS: ANOVA		*P* < 0.0001	*P* < 0.001	NS	*P* < 0.001
Glucose metabolism status					
Normal	1501/2243	52.7 ± 6.5^∗^	51.4 ± 8.4 ^#^	50.2 ± 8.3	46 ± 10.6^+^
Prediabetes	276/306	50.6 ± 8^∗∗^	48.3 ± 10.1^##^	50.6 ± 9.1	43.7 ± 11.5
Unknown diabetes	140/103	49.9 ± 8.4	46 ± 11.1	49.8 ± 9.3	43.7 ± 11.8
Known diabetes	245/233	48.8 ± 8.8	44.6 ± 11.5	50.9 ± 9.3	44.5 ± 11.8
SS: ANOVA		*P* < 0.0001	*P* < 0.0001	NS	*P* < 0.0001

PCS 12, the physical summary component score. MCS 12, the mental summary component score. Pre-diabetes, IFG (impaired fasting glucose) and/or ITG (impaired glucose tolerance). Results expressed as mean ± SD; M/W, (men/women); NS (nonsignificant).

SS: ANOVA evaluates intragroup differences for age, BMI, educational level, and glucose metabolism status.

^∗^ *P* < 0.001, ^#^ *P* < 0.001 denote differences between normal and all of the groups (prediabetes, UKDM, and KDM).

^∗∗^ *P* < 0.02 denote pre-diabetes versus known diabetes.

^##^ *P* < 0.001 denote pre-diabetes versus known diabetes.

^+^ *P* < 0.03 denote normal versus pre-diabetes.

**Table 2:** Odds ratios for PCS 12 and MCS 12 scores lower than the median (Model 1) adjusted for age (Model 2), and adjusted for age, educational level, and BMI, all stratified by gender (Model 3).

SCHOM	PCS 12			MCS 12		
	OR	95% IC	*P* value	OR	95% IC	*P* value
Model 1						
Normal	1			1		
Prediabetes						
Men	1.54	1.19–1.99	**0.001 **	0.92	0.7–1.2	0.534
Women	1.72	1.34–2.19	**0.000**	1.24	0.97–1.59	0.089
UNKDM						
Men	1.59	1.12–2.25	**0.09 **	1.16	0.82–1.65	0.404
Women	2.09	1.38–3.17	**0.01**	1.31	0.87–1.99	0.196
KDM						
Men	2.45	1.85–3.25	**0.000 **	0.81	0.61–1.08	0.152
Women	3.11	2.29–4.21	**0.000**	1.04	0.79–1.37	0.761

Model 2						
Normal	1			1		
Prediabetes						
Men	1.25	0.96–1.63	0.105	1.02	0.78–1.35	0.878
Women	1.33	1.03–1.71	**0.029**	1.33	1.03–1.72	**0.027**
UNKDM						
Men	1.23	0.86–1.77	0.251	1.32	0.92–1.89	0.135
Women	1.46	0.95–2.23	0.085	1.46	0.96–2.23	0.080
KDM						
Men	1.83	1.36–2.46	**0.000 **	0.94	0.69–1.27	0.692
Women	2.15	1.56–2.95	**0.000**	1.16	0.87–1.55	0.321

Model 3						
Normal	1			1		
Prediabetes						
Men	1.17	0.89–1.53	0.265	1.05	0.80–1.39	0.722
Women	1.10	0.84–1.43	0.487	1.21	0.93–1.57	0.156
UKDM						
Men	1.07	0.74–1.54	0.719	1.35	0.93–1.95	0.113
Women	1.12	0.73–1.74	0.601	1.33	0.86–2.05	0.197
KDM						
Men	1.62	1.2–2.19	**0.002 **	0.95	0.7–1.30	0.765
Women	1.75	1.26–2.43	**0.001**	1.01	0.75–1.37	0.927

PCS 12, the physical component summary score. MCS 12, the mental component summary score. SCHOM, status of the carbohydrate metabolism. “Prediabetes” includes impaired fasting glucose and impaired glucose tolerance. UNKDM, unknown diabetes mellitus. KDM, known diabetes mellitus.
